# HIV incidence and associated risk factors in female spouses of men who inject drugs in Pakistan

**DOI:** 10.1186/s12954-021-00497-1

**Published:** 2021-05-08

**Authors:** Jenny Iversen, Salman ul H Qureshi, Malika Zafar, Machteld Busz, Lisa Maher

**Affiliations:** 1 Viral Hepatitis Epidemiology and Prevention Program, The Kirby Institute, UNSW Sydney, Sydney, NSW 2052 Australia; 2 Nai Zindagi Trust, 37-38, Beverly Center, Blue Area, Islamabad Capital Territory, 46000 Pakistan; 3 Stichting Mainline, Frederik Hendrikstraat 111-115, 1052 HN Amsterdam, The Netherlands

**Keywords:** Drug use, HIV prevention, Key and vulnerable populations, Women, Pakistan

## Abstract

**Introduction:**

Female sexual partners of men who inject drugs (MWID) living with HIV are at risk of HIV transmission. HIV prevalence estimates among non-drug using female sex partners of MWID are scarce, with no studies documenting HIV incidence. We investigated HIV prevalence and incidence among female spouses of MWID registered at Nai Zindagi Trust (NZT), Pakistan, between 2012 and 2019.

**Methods:**

NZT registration and service provision data for female spouses who participated in HIV testing and counselling calculated HIV prevalence and incidence using the person years (PY) method. Cox proportional hazards models identified factors associated with incident infection.

**Results:**

Overall HIV prevalence among female spouses of MWID was 8.5%. Among 3478 HIV-negative female spouses, 109 incident infections were observed, yielding an incidence rate of 1.5/100PY (95% CI 1.2–1.8). Independent predictors of incident infection were registration in Punjab province (AHR 1.73 95% CI 1.13–2.68, *p* = 0.012) and 1–5 years of education (AHR 1.89 95% CI 1.22–2.93, *p* = 0.004). Knowledge of HIV at registration was protective against infection (AHR 0.51, 95% CI 0.26–0.99, *p* = 0.047), along with a MWID spouse who had initiated antiretroviral therapy (ART) (AHR 0.25, 95% CI 0.16–0.38, *p* < 0.001), while incident infection was inversely associated with number of children (≥ 5 children AHR 0.44 95% CI 0.22–0.88, *p* = 0.022).

**Conclusions:**

Additional efforts are needed to reduce HIV transmission among female spouses of MWID, including targeted provision of HIV education and access to HIV screening. Interventions that target MWID are also required, including evidence-based drug treatment and access to ART, including support to maximize adherence. Finally, consideration should be given to making HIV pre-exposure prophylaxis available to female spouses at high risk of HIV transmission, particularly young women and those whose husbands are not receiving, or have difficulty adhering to, ART.

## Introduction

Globally an estimated 18% of people who inject drugs (PWID) are living with human immunodeficiency virus (HIV), including 19% in South Asia [1]. It is therefore concerning that Pakistan’s National AIDS Control Program 2016/17 Integrated Biological and Behavioural Surveillance reported country-level HIV prevalence among PWID at 38% [2]. Similarly, among PWID attending Nai Zindagi Trust (NZT), a non-government agency providing services to people and communities affected by drug use, HIV prevalence among PWID in June 30, 2019, was double the global and regional prevalence estimate at 36% [3]. Notwithstanding the concentrated epidemic of HIV infection among PWID in Pakistan, UNAIDS reports indicate a decline in HIV prevalence among PWID in recent years, from 27% in 2011 to 21% in 2019 [4].

While HIV infection in Pakistan is concentrated among PWID, gay and bisexual men, sex workers, and transgender people [5], a recent iatrogenic outbreak of HIV among ~ 600 children in Larkana District (Sindh province) highlights the potential for transmission beyond these key populations [5, 6]. A recent systematic review found that women are a growing population of people living with HIV infection, particularly women who live in rural settings and in poverty [7]. Among the estimated 25,000 new HIV per annum infections occurring in Pakistan, 6700 (27%) occur among women [4]. It is well documented that sexual partners of people with HIV who have unsuppressed viral load are at risk of HIV infection, with an estimated HIV transmission risk of 138 and 8 per 10,000 exposures for receptive anal and penile-vaginal intercourse respectively [8]. However, female sexual partners of HIV-positive men who inject drugs (MWID) remain an understudied population, with only nine studies, presenting HIV prevalence estimates among non-drug using female sex partners of HIV-positive MWID globally over the past two decades (since 2000) [9–17]. Among these studies, HIV prevalence varied from a high of 45% in Manipur, India, [10, 18] and 35% in Kohtla-Järve, Estonia, [9] to a low of 3% in Tehran, Mashhad and Shiraz, Iran [17]. Less is known about incident HIV infection among this group and no studies presenting HIV incidence among non-drug using female sexual partners of HIV-positive MWID were identified.

An estimated 100,000 PWID reside in Pakistan’s four provinces (Punjab, Sindh, Balochistan and Khyber Pakhtunkhwa) [19]. The vast majority (99%) of PWID in Pakistan are men and around one-third are married [2]. Less than one quarter report using condoms with their spouse or regular sex partner [19]. This study aimed to investigate HIV prevalence and incidence among female spouses of MWID who were registered at NZT between March 2012 and December 2019 and who received care and support services, including HIV testing and counselling (HTC).

## Methods

NZT provides harm reduction and HIV prevention, care and support services to PWID in 38 Districts in Pakistan, with services provided in 29 Districts covering all four provinces at the time of this study. With verbal consent of husbands, female spouses of MWID were identified and contacted by female outreach workers. Consenting female spouses were provided with HIV prevention, care and support services, including voluntary HTC. In the early years of the project (March 2012–December 2013), all female spouses were eligible for registration; however, HIV testing and counselling were restricted to spouses of HIV-positive MWID from 2014. Female spouses were all legally married. All individuals were registered using a unique registration code that enabled linkage of multiple occasions of service provision and linkage to MWID spouses. HIV testing was offered to female spouses of MWID who were identified as HIV positive. Female spouses that tested HIV negative were provided with HIV prevention and counselling services, with follow up HTC encouraged at 3 monthly intervals. The Institutional Review Board at NZT granted ethical approval for this study.

### Databases

The current study drew on data from comprehensive NZT registration and service provision databases. Data collected at registration among female spouses included geographic location, birth year, nationality, education, source of income, age at first marriage, number of children, pregnancy status, sexual history and condom use and history of surgical procedures and sexually transmissible diseases, and injection drug use. Female spouses were also asked if they had ever heard of HIV/AIDS, hepatitis B and hepatitis C. Additional datasets collected details of all subsequent service provision, including HTC and test results among female spouses and harm reduction service provision and antiretroviral therapy (ART) initiation among MWID spouses.

### HIV serological testing

Voluntary HIV testing was conducted using rapid diagnostic tests according to Pakistan’s National HIV testing guidelines, with two serial rapid diagnostic tests (RDTs) conducted until December 2016, and three RDTs from January 2017. Serial rapid test platforms were Alere DetermineTM HIV-1/2 Ag/Ab Combo (Sensitivity > 99%, Abbott Japan Co Ltd, Tokyo, Japan), Uni-Gold HIV (Specificity > 99%, Trinity Biotech PLC, Bray, Ireland), SD Bioline HIV-1/2 3.0 (Sensitivity > 99%, Specificity > 98%, Standard Diagnostics Inc, Kyonggi-do, Korea). As per National HIV testing guidelines, where an indeterminate test result was obtained, repeat testing was offered after 15 days.

### Analysis dataset

The analysis dataset was created using data collected from female spouses of MWID attending NZT at registration (March 2012–December 2018) and subsequent service provision (March 2012–March 2019). Service provision data (condom procurement and date of ART initiation) among NZT registered MWID was merged into the female spouse analysis dataset. The dataset was restricted to female spouses of HIV-positive MWID and to those who had participated in HTC. Baseline HIV prevalence among female spouses of HIV-positive PWID was calculated among the group with a minimum of one HIV test result. HIV incidence was calculated among the group who were HIV negative at first test and who had at least one subsequent HIV test result recorded. A small proportion (< 0.5%) of HIV test results were indeterminate and these records were removed from the analysis dataset to avoid the risk of misclassifying an incident infection.

### Data analysis

The primary outcome was HIV seroconversion among female spouses of HIV-positive MWID. The date of seroconversion was taken as the midpoint between date of last HIV negative and first HIV-positive test. Follow-up time was estimated using the person years (PY) method, where the follow-up time was the difference between date of initial HIV-negative test and date of HIV infection or date of last negative test for those who did not acquire HIV infection. Records were censored at the estimated date of HIV infection or the date of last HIV test among those who remained HIV negative. HIV incidence was determined using Kaplan–Meier methods and cox proportional hazards analyses determined factors associated with HIV incident infection. The adjusted model considered all factors where *p* < 0.10, and the final model included only factors that remained significant at *p* ≤ 0.05, with hazard ratios (HRs) and corresponding 95% confidence intervals (CIs) obtained. All analyses were conducted using STATA software version 14 (Stata Corporation, College Station, TX, USA).

## Results

**Fig. 1:**
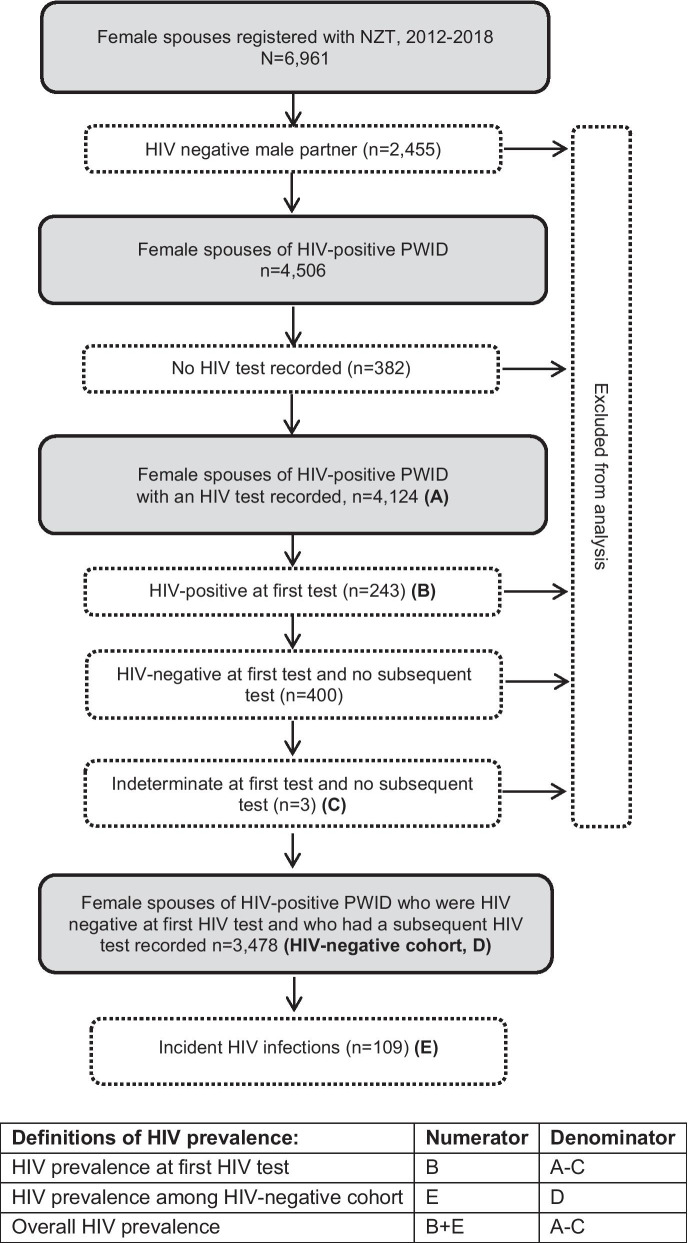
Creation of HIV-negative cohort using HIV test results of female spouses of male HIV-positive PWID between 2012 and 2018 and definitions of HIV prevalence

A total of 6961 female spouses of MWID registered with NZT were identified and registered with NZT between March 2012 and December 2018. Around one third of female spouses were unable to be contacted, primarily due to separation, including geographic separation. After excluding 2012–2013 records for female spouses with husbands who were HIV negative, 4506 female spouses remained, with the majority (4124, 92%) participating in voluntary HTC. Three female spouses had an indeterminate first test result and no subsequent HIV test, while 243 (6%) women were identified as HIV-positive at the time of their first test, including 32 women who were pregnant. Female spouses with indeterminate or HIV-positive test results at first test were excluded from the analysis dataset, along with a further 400 (10%) spouses who were HIV negative at first test who had no subsequent HIV test and were considered lost to follow up. Thus, 3478 HIV negative female spouses of HIV-positive MWID who had > 1 HIV test were retained in the study dataset, with a total 24,224 test records (Fig. 1). Among this group, a median of five (Interquartile range [IQR] 3–9) HIV tests were conducted, and the median time between tests was 3.4 months (IQR 3.0–4.9 months).

More than half (2197, 63%) of the female spouses who were HIV negative at first test were registered in one of 15 cities in Punjab province, with a further one third (1213, 35%) registered in one of 11 cities in Sindh province. The remaining sample were registered in Quetta or Turbat in Balochistan province (30, 1%) and Peshawar city in Khyber Pakhtunkhwa province (38, 1%). One woman was Afghani, while all remaining women were Pakistani. The median age at registration was 30 years (IQR 26–36 years), while the median age at last HIV test was 32 years (IQR 28–38 years). The median age at first marriage was 18 years (IQR 16–21 years) and the median number of children at registration was three (IQR 2–4). The majority of female spouses had little or no education (2957, 85%) and had no awareness of HIV at registration (2902, 83%), and most worked in the textile industry (1787, 51%) or had no employment (1354, 39%). A minority (15, < 0.5%) of female spouses reported a lifetime history of illicit drug injection and just over half (1774, 51%) had a male spouse who had initiated ART prior to date of HIV infection or last HIV negative test.

### Incident HIV infection

Among female spouses of HIV-positive MWID who were HIV negative at first HIV test and received > 1 non-indeterminate HIV test result between March 2012 and March 2019, 109 incident HIV infections were observed, yielding an incidence rate of 1.5 per 100 PY (95% CI = 1.2–1.8). Despite high HIV prevalence at first HIV test in Khyber Pakhtunkhwa (12/52, 23%) and Balochistan provinces (9/41, 22%, Table 1), there were no incident HIV infections observed in Khyber Pakhtunkhwa and only one incident infection observed in Balochistan province, where cumulative incidence was 3%. In Punjab and Sindh provinces, HIV prevalence at first test was 6% (152/2607) and 5% (70/1421), respectively. After inclusion of incident infections, HIV prevalence at last test was 9% (232/2607) in Punjab province and 7% (98/1421) in Sindh province.

**Table 1:** HIV prevalence at first HIV test, last HIV test and cumulative HIV incidence among female spouses by province, 2012–2019

Geographic location	HIV prevalence at first test Number (%)	Cumulative HIV incidence Number (%)	HIV prevalence last HIV test Number (%)
Total	243/4121 (5.9)	109/3478 (3.1)	352/4121 (8.5)
Balochistan	9/41 (22.0)	1/30 (3.3)	10/41 (24.4)
Khyber Pakhtunkhwa	12/52 (23.1)	0/38 (0.0)	12/52 (23.1)
Punjab	152/2607 (5.8)	80/2197 (3.6)	232/2607 (8.9)
Sindh	70/1421 (4.9)	28/1213 (2.3)	98/1421 (6.9)

**Fig. 2:**
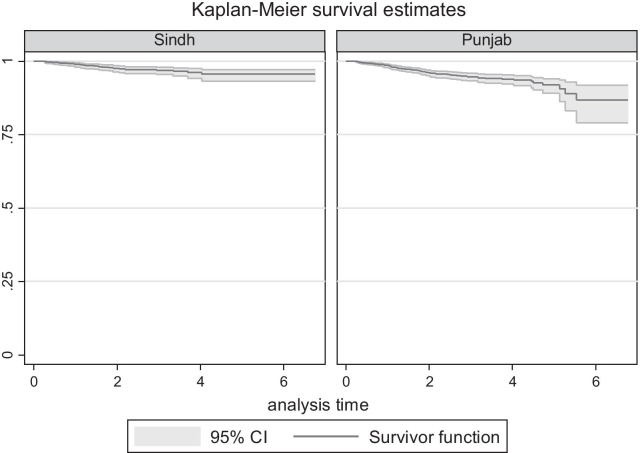
Kaplan–Meier survival graphs by Province (log-rank-test, *p* = 0.011). **Note**: Kaplan-Meier graph showing HIV incident infection among spouses of HIV positive men who inject drugs in Sindh and Punjab. Khyber Pakhtunkhwa and Balochistan not shown due to small sample size

In univariable analysis (Table 2), HIV incident infection was significantly lower among female spouses with five or more children compared to those with no children, among women who were aware of HIV at the time of registration and those with spouses who had initiated ART. In contrast, HIV incident infection was significantly higher among female spouses who were registered in Punjab province (Fig. 2), women aged < 30 years and those with 1–5 years of education compared to those with no education. In multivariable analysis, independent predictors of incident HIV infection were registration in Punjab province (Adjusted Hazard Ratio [AHR] = 1.73, 95% CI 1.13–2.68, *p* = 0.012) and 1–5 years of education relative to those with no education (AHR = 1.89, 95% CI 1.22–2.93, *p* = 0.004). Knowledge of HIV at registration was identified as protective against incident HIV infection (AHR = 0.51 95% CI 0.26–0.99, *p* = 0.047), along with having a MWID spouse who had initiated ART (AHR = 0.25 95% CI 0.16–0.38, *p* < 0.001). The odds of HIV incident infection was inversely associated with the number of children reported by female spouses (1–2 children AHR = 0.60, 95% CI 0.33–1.09, *p* = 0.095; 3–4 children AHR = 0.55, 95% CI 0.30–1.00, *p* = 0.053; ≥ 5 children AHR = 0.44, 95% CI 0.22–0.88, *p* = 0.022).

**Table 2:** Cumulative incidence, incidence rate per 100 person years, and unadjusted and adjusted hazard ratios among female spouses of male HIV-positive PWID by demographic characteristics, HIV risk behaviour and knowledge

Characteristic	Cumulative incidence Number (%)	Incidence density per 100 person years (95% CI)	Unadjusted hazard ratio (95% CI)	*p* value	Adjusted hazard ratio (95% CI)	*p* value
Total	*N* = 109/3478 (3.1)	1.5 (1.2–1.8)				
*Province*						
Sindh	28/1213 (2.3)	1.0 (0.7–1.5)	1.0 (Reference)	–	1.0 (Reference)	–
Punjab	80/2197 (3.6)	1.8 (1.4–2.2)	1.74 (1.13–2.67)	0.012	1.73 (1.13–2.68)	0.012
Khyber Pakhtunkhwa	0/38 (0)	–	–	–	–	–
Balochistan	1/30 (3.3)	1.5 (0.2–10.3)	1.44 (0.20–10.6)	0.978	0.98 (0.13–7.2)	0.983
*Age group (tertiles)*						
≥ 36 years	26/1168 (2.3)	1.1 (0.7–1.5)	1.0 (Reference)	–		
30–35 years	37/1122 (3.3)	1.6 (1.2–2.2)	1.55 (0.94–2.26)	0.086		
< 30 years	46/1188 (3.9)	1.9 (1.4–2.5)	1.79 (1.10–2.92)	0.019		
*Age at first marriage (tertiles)*						
10–17 years	40/1220 (3.3)	1.5 (1.1–2.0)	1.0 (Reference)	–		
18–20 years	37/1373 (2.7)	1.3 (0.9–1.8)	0.86 (0.55–1.34)	0.505		
≥ 21 years	32/885 (3.6)	1.9 (1.3–2.7)	1.25 (0.79–2.00)	0.341		
*Children*^*a*^						
None	15/322 (4.7)	2.5 (1.5–4.2)	1.0 (Reference)	–	1.0 (Reference)	–
1–2 children	41/1273 (3.2)	1.5 (1.1–2.1)	0.61 (0.34–1.10)	0.100	0.60 (0.33–1.09)	0.095
3–4 children	36/1214 (3.0)	1.4 (1.0–2.0)	0.56 (0.31–1.02)	0.060	0.55 (0.30–1.00)	0.053
≥ 5 children	17/669 (2.5)	1.1 (0.7–1.8)	0.45 (0.22–0.90)	0.024	0.44 (0.22–0.88)	0.022
*Education*^*a*^						
None	71/2441 (2.9)	1.4 (1.1–1.7)	1.0 (Reference)	–	1.0 (Reference)	–
1–5 years	29/516 (5.6)	2.7 (1.9–3.8)	1.92 (1.25–2.96)	0.003	1.89 (1.22–2.93)	0.004
6–10 years	7/350 (2.0)	1.0 (0.5–2.1)	0.74 (0.34–1.61)	0.451	0.86 (0.39–1.89)	0.707
High school graduate	2/171 (1.2)	0.6 (0.1–2.3)	0.43 (0.11–1.76)	0.241	0.54 (0.13–2.25)	0.399
*Source of income*^*a*^						
None	48/1354 (3.6)	1.7 (1.3–2.2)	1.0 (Reference)	–		
Textiles	50/1787 (2.8)	1.4 (1.0–1.8)	0.82 (0.55–1.22)	0.331		
House main/cleaner	6/155 (3.9)	1.6 (0.7–3.6)	0.98 (0.42–2.30)	0.967		
Beautician	3/41 (7.3)	3.4 (1.1–10.6)	2.00 (0.62–6.41)	0.246		
Other	2/141 (1.4)	0.7 (0.2–2.7)	0.41 (0.10–1.67)	0.212		
*Ever surgical procedure*^*a*^						
No	93/2915 (3.2)	1.5 (1.2–1.9)	1.0 (Reference)	–		
Yes	16/563 (2.8)	1.4 (0.8–2.2)	0.89 (0.52–1.52)	0.673		
*Ever STI treatment*^*a*^						
No	108/3318 (3.3)	1.6 (1.3–1.9)	1.0 (Reference)	–		
Yes	1/160 (0.6)	0.3 (0.04–2.0)	0.18 (0.02–1.27)	0.086		
*Condom used at last sexual intercourse*^*a*^						
No	97/2981 (3.3)	1.6 (1.3–1.9)	1.0 (Reference)	–		
Yes	12/497 (2.4)	1.2 (0.7–2.1)	0.76 (0.41–1.38	0.360		
*Ever injected drugs*^*a*^						
No	108/3463 (3.1)	1.5 (1.2–1.8)	1.0 (Reference)	–		
Yes	1/15 (6.7)	3.0 (0.4–20.9)	1.96 (0.27–14.0)	0.505		
*Heard about HIV*^*a*^						
No	99/2902 (3.4)	1.7 (1.4–2.0)	1.0 (Reference)	–	1.0 (Reference)	–
Yes	10/576 (1.7)	0.8 (0.4–1.5)	0.48 (0.25–0.92)	0.026	0.51 (0.26–0.99)	0.047
*Male spouse average condoms received per annum (tertiles)*						
< 4	27/1109 (2.4)	1.4 (0.9–2.0)	1.0 (Reference)	–		
5–15	36/1113 (3.2)	1.6 (1.2–2.3)	1.21 (0.73–1.99)	0.460		
> 15	46/1256 (3.7)	1.5 (1.1–2.0)	1.10 (0.68–1.77)	0.703		
*Male spouse initiated ART*^*b*^						
No	82/1704 (4.8)	2.7 (2.1–3.3)	1.0 (Reference)		1.0 (Reference)	–
Yes	27/1774 (1.5)	0.6 (0.4–0.9)	0.24 (0.15–0.37)	< 0.001	0.25 (0.16–0.38)	< 0.001

*STI* Sexually transmissible infection, *HIV* Human immunodeficiency virus, *ART* antiretroviral therapy

^a^At baseline

^b^Prior to censorship

## Discussion

In this large sample of female spouses of HIV-positive MWID who were tested for HIV (4121), overall HIV prevalence was 8.5% compared with 0.1% among the general population aged 15–49 years in 2019 [4]. Prevalent HIV infection was particularly high in Khyber Pakhtunkhwa (23% of 52 women) and Balochistan provinces (24% of 41 women), with 95% (21/22) of these infections identified at first HTC contact with NZT. Among HIV negative female spouses of HIV-positive MWID (3478), HIV incidence was 1.5 per 100 PY, with significantly higher incident infection observed in Punjab province compared to Sindh province. Consistent with findings from the 2017/18 Demographic and Health Survey where HIV health literacy and a woman’s ability to negotiate sexual relations with her husband varied by region [20], findings indicate risk of HIV infection among female spouses of HIV-positive MWID in Pakistan is subject to significant geographic variation.

Knowledge of HIV at registration was protective against HIV infection in female spouses of HIV-positive MWID. It is therefore alarming that awareness of HIV is low in Pakistan, with an estimated 68% of women nationally [20] and 83% of women in this study reporting no awareness of HIV at first HTC. Given that only a minority (15%) of women in this study had more than five years of formal education, the provision of community HIV education programs that target women, particularly younger women and those with low levels of formal education appears warranted. This study found that HIV prevalent infection at first HIV test was 6%, although prevalent infection declined to 3% among women who were provided with ongoing HTC. Counselling and testing can reduce HIV related-risk through modification of sexual behaviour in low income countries [21], and it is therefore likely that HTC provided by NZT (among both MWID and their female spouses) contributed a prevention benefit.

Self-reported lifetime injection drug use among female spouses of MWID was low (0.4%), although women were not specifically asked about injections received from a community provider, where services were provided by either a qualified doctor or an unqualified “quack”. An earlier study of female spouses of MWID in Lahore and Faisalabad, consisting of 102 women recruited consecutively by randomly selecting their husband’s names from NZT records and four female sex workers identified by local NGOs as having long-term PWID sexual partners, found that 23% used drugs, including 19% who reported injection drug use [22]. In this study, most of the women who reported injection drug use (17/18) reported receiving combined injections of diazepam and pheniramine from community providers (“doctor/quack”), with only one woman (1%) reporting administering her own injections. Average monthly injections among this group were 20 (range 5–60 injections). Most were not aware of how their injections were prepared or whether sterile equipment or techniques were used. The authors concluded that female spouses of MWID in this setting were at moderate risk of acquiring HIV from their husbands via infrequent but unprotected sexual intercourse given underlying HIV prevalence of 21% among MWID in Pakistan at the time. However, among female spouses who reported injecting drugs, injection from community providers represented an additional potential exposure to HIV [22].

In this study, 89% of women with prevalent HIV infection and 86% of the women who acquired incident HIV infection had children, and 32 women who tested HIV-positive were pregnant at the time of registration. During the period of this study, testing guidelines in Pakistan provided for HIV testing of the youngest child of HIV-positive mothers and only provide additional testing for siblings where the youngest child was identified as HIV positive. However, testing guidelines were changed and currently provide HIV testing for siblings regardless of HIV status of the youngest child. Among the 320 HIV-positive women who had children or who were pregnant at registration, 47% (151) had children who were subsequently tested for HIV. Among the 353 children tested, 10% (34) were identified as HIV-positive (data not shown), indicating substantial risk of mother to child HIV transmission among female spouses of HIV-positive MWID. However, exposure to HIV from community sources, including use of contaminated medical equipment, cannot be ruled out [5, 6, 23, 24]. It is concerning that the children of only half of HIV-positive women with children were tested, indicating a need to scale up HIV testing among children at risk of infection and identify strategies to retain pregnant women in care and support services.

HIV antiretroviral therapy can achieve full or partial virological suppression and reduce transmission risk among serodiscordant couples [25]. Although this study identified uptake of ART among HIV-positive MWID as protective against HIV incident infection, 27 HIV transmissions were documented among females with spouses who had initiated ART. In three of these cases, MWID had initiated ART less than 6 months prior to the estimated date of HIV seroconversion among their female partners and may not have achieved virological suppression. However, a previous study conducted among HIV-positive MWID in Karachi (Sindh Province, Pakistan) concluded that ART adherence was low in this group, with only 19% of MWID considered adherent, defined as < 4 missed ART doses per month [26], with non-adherence predominantly due to MWID forgetting to take their medication. In this study, family support and social cohesion were identified as factors contributing to ART adherence in MWID [26]. It is therefore essential that both MWID and their female spouses are aware that adherence to daily ART regimes over an extended time period is required to achieve viral suppression and reduce transmission risk among sero-discordant couples [27], as well as being supported to achieve and maintain high adherence.

This study has some limitations. Although none of the female spouses reported sex work as their main source of income, women were not specifically asked whether they engaged in sex work. As such, we were unable to determine whether any of these women engaged in sex work which potentially placed them at additional risk of HIV transmission. Similarly, women were not specifically asked about injections administered by community providers, which also potentially provided an additional exposure to HIV. Previous work suggests that MWID registered with NZT are likely to be broadly representative of MWID in Pakistan, given the low number of women who inject drugs and the high proportion of NZT registered MWID (≥ 60%) compared with PWID population size estimates [22]. Not all female spouses were able to be contacted or registered with NZT. This was predominantly due to separation of partners, including geographic separation [16]. This group of female spouses, who were unable to be contacted, is considered to have a low risk of intimate partner HIV transmission given they were separated.

A multi-pronged approach is required to reduce the risk of HV transmission among female spouses of HIV-positive MWID. The approach requires targeting of PWID through scale up of existing harm reduction programs to further prevent HIV transmission among this key population [28]. Consideration should also be given to providing targeted programs, including social network interventions to disseminate awareness and/or testing [29–31], to women who inject drugs, as well as women who receive injections from community providers. There is also a need for evidence-based drug treatment, not currently available in this setting. Opioid agonist therapy (OAT) has also been shown to reduce HIV incidence (primarily by reducing the frequency of injection) in PWID dependent on opioids [32]. The introduction of OAT for both men and women who inject drugs in this setting would complement the existing comprehensive suite of harm reduction interventions already provided to PWID in Pakistan by NZT and provide an important tool to prevent primary and secondary HIV transmission.

It is also necessary to scale up initiation of ART among MWID, as well as support adherence, and to ensure that both men and women understand that viral suppression is not immediate or guaranteed. In this regard, MWID should be encouraged to use condoms and to allow women to participate in the negotiation of sexual relations. As previously mentioned, a community-based HIV awareness program targeting women, particularly young women and those with low literacy, is warranted and should be accompanied by access to HIV testing for those at risk of infection. Finally, HIV pre-exposure prophylaxis (PREP) should be considered for female spouses of HIV-positive MWID who are at high risk of HIV transmission, particularly women living in Punjab province, younger women with fewer children and those with husbands who have not initiated, or have difficulty adhering to ART.

## Conclusions

To our knowledge, this study is the first to document HIV incidence among female spouses of MWID in Pakistan and internationally. Among women who were HIV negative at first HIV test, 3.1% (109/3478) subsequently tested HIV-positive for an HIV incidence rate of 1.5 per 100 PY. However, more than double this number of women (243 female spouses of HIV-positive MWID) tested HIV-positive at their first HTC encounter with NZT. Overall HIV prevalence among female spouses was 8.5% compared with 0.1% among the general population aged 15–49 years in Pakistan. Our results indicate that more needs to be done; and support the need for early intervention to prevent HIV transmission among women who are married to MWID in this setting.

## Data Availability

None.
